# Health Professionals’ Perceptions about Prostate Cancer—A Focus Group Study

**DOI:** 10.3390/cancers16173005

**Published:** 2024-08-29

**Authors:** Catarina Leitão, Marta Estrela, Luís Monteiro, Margarida Fardilha, Maria Teresa Herdeiro, Fátima Roque

**Affiliations:** 1 Department of Medical Sciences, Institute of Biomedicine (iBiMED), University of Aveiro, Campus Universitário de Santiago, 3810-193 Aveiro, Portugal; mestrela@ua.pt (M.E.); mfardilha@ua.pt (M.F.); teresaherdeiro@ua.pt (M.T.H.); 2 Department of Social, Political and Territorial Sciences, University of Aveiro, 3810-193 Aveiro, Portugal; 3 Centre for Health Studies and Research, University of Coimbra, 3004-512 Coimbra, Portugal; 4 Health Sciences Research Centre, University of Beira Interior (CICS-UBI), Av. Infante D. Henrique, 6200-506 Covilhã, Portugal; 5 CINTESIS@RISE—Centre for Health Technology and Services Research, Faculty of Medicine, University of Porto, 4200-450 Porto, Portugal; monteiroluis@ua.pt; 6 Department of Medical Sciences, University of Aveiro, Campus Universitário de Santiago, 3810-193 Aveiro, Portugal; 7 Biotechnology Research, Innovation and Design for Health Products (BRIDGES), Research Laboratory on Epidemiology and Population Health, Polytechnic of Guarda, Avenida Dr. Francisco Sá Carneiro, 6300-559 Guarda, Portugal

**Keywords:** perceptions, knowledge, attitudes, health professionals, risk factors, lifestyle, prostate cancer, screening/diagnosis, treatment

## Abstract

**Simple Summary:**

Prostate cancer (PCa) is a major health issue among men, with risk factors such as age, family history, genetics, and race being well known. However, the impact of lifestyle choices has often been overlooked. This study aimed to understand health professionals’ perceptions of PCa risk factors to design a questionnaire applicable to the male population. Three focus groups were conducted to discuss topics, such as the impact of diet, physical activity, alcohol consumption, smoking, sexual health, and screening and treatment methods. Although healthy lifestyles can generally improve PCa outcomes, no specific lifestyle change has been identified as a preventive measure, particularly for older adults. Lycopene, present in tomatoes, was highlighted for its antioxidant benefits. This study demonstrates the necessity for additional research on how lifestyle affects PCa and emphasizes the importance of health professionals in encouraging the adoption of healthier habits. It also suggests exploring non-invasive screening through urine and innovative treatment methods to reduce false positives and side effects, respectively.

**Abstract:**

Prostate cancer (PCa) accounts for 20% of new cancer cases and 10.5% of cancer-associated mortality in Portugal. Associated risk factors include advanced age, family history, genetic alterations, and race/ethnicity. However, the role of lifestyle factors is often underestimated. To explore health professionals’ perceptions of PCa risk factors, a qualitative study with three focus groups (FG), with a total of twenty-one general practitioners and urologists, was conducted via videoconference between February and April 2023. Seven themes emerged, including general perceptions of PCa; PCa risk factors; nutritional impact; the role of physical activity; alcohol consumption and smoking; sexual activity and sexually transmitted diseases roles in PCa; and screening, diagnosis, and treatment methods. Despite agreeing that healthy lifestyles could promote better PCa outcomes and quality of life, participants did not specify any lifestyle factors that could promote or prevent this disease, posing challenges to lifestyle changes, particularly among older adults. Non-invasive screening methods, such as biomarkers and alternative treatments, are crucial for future research. This study underscores the need for further investigation into the correlation of lifestyle factors with PCa and highlights the necessity of health professionals in encouraging their patients to adopt healthier lifestyles, while offering important insights into awareness, prevention, and alternative screening, diagnosis, and treatment methods, which could help reduce false positives and treatment side effects.

## 1. Introduction

Prostate cancer (PCa) is the most prevalent cancer and the third deadliest cancer among men in Europe [1]. In Portugal, PCa accounts for 20% of the estimated new cancer cases and 10.5% of cancer-associated mortality [1]. Since the implementation of prostate-specific antigen (PSA) as a diagnostic tool for PCa, most countries have registered a significant increase in PCa incidence [2]. However, many patients with increased PSA levels do not present any symptoms, leading to unnecessary treatment [3]. This highlights the need for more precise diagnostic strategies and a deeper understanding of PCa risk factors to avoid overdiagnosis and overtreatment, which may be achieved by exploring potential risk factors that are not yet well established.

Several risk factors are associated with PCa including advanced age, ethnicity, genetic alterations, and family history. Additionally, growing evidence suggests that a healthy lifestyle may improve overall clinical outcomes and quality of life [2,4,5,6,7,8,9]. However, after diagnosis, the number of men who change their lifestyle habits, such as diet and physical activity (PA), is relatively low [10,11]. A recent study evaluating the reasons for men not changing their habits reported that most men believed that external factors such as advanced age and genetics were the main sources of PCa [12]. This was supported by a previous qualitative study that stated that PCa patients underestimated lifestyle habits as risk factors [13]. Moreover, a study [14] evaluating the nutritional status of men diagnosed with PCa in Portugal showed a high prevalence of obesity, excessive body weight, and abdominal fat. Additionally, their diets were predominantly deficient in vegetables, nuts, selenium, lycopene, and other phytochemically rich foods. Since almost one-third of cancers are preventable through healthy diets and lifestyles [15], PCa diagnosis may be used as a teachable moment to raise awareness about the role of lifestyle factors in the progression of this disease.

While research has focused on patient behavior and overall PCa perception [16,17,18], there is limited understanding of how health professionals perceive their risk factors. Health professionals play a pivotal role in the early detection of PCa and in patient education. Therefore, their perceptions, knowledge, and attitudes can significantly influence patient outcomes. Understanding these perceptions may help to identify gaps in knowledge, improve communication strategies, and ultimately enhance patient care.

The main goal of this study was to explore and understand health professionals’ perceptions of PCa. To achieve this goal, a focus group (FG) study was conducted to identify key themes and insights that may inform future educational and intervention strategies. This type of study can be an enlightening research tool, as it highlights frequently unexplored dimensions of a problem, including those that are unanticipated [19]. Our study had three specific aims: (i) to explore health professionals’ beliefs about the most significant PCa risk factors; (ii) to identify, through their professional experience, the barriers to lifestyle modification among their patients; and (iii) to understand the educational gaps of health professionals regarding PCa.

This study provides valuable insights into how healthcare professionals view and manage PCa risk factors, thereby contributing to more effective prevention and treatment strategies.

## 2. Materials and Methods

### 2.1. Ethical Approval

This study was performed in line with the principles of the Declaration of Helsinki. This study obtained ethical approval from the Ethics Committee of Portugal’s Center Regional Health Administration (Administração Regional de Saúde do Centro IP/ARS-Centro) (registry no. 53/2022), and it follows the EU Regulation 2016/679 of the European Parliament and Council of 27 April 2016, on the protection of individuals regarding the processing of personal data and the free movement of such data, which repeals Directive 95/46/EC (GDPR).

### 2.2. Setting and Sample

The FG sessions were conducted with general practitioners (GPs) and urologists from the central region of Portugal. The National Health System has 8554 GPs and 468 urologists working in this region [20]. For this qualitative study, participants were invited through a snowball sampling strategy via e-mail or face-to-face meetings.

### 2.3. Study Design

A qualitative study with FGs was designed to explore the perceptions and experiences of GPs and urologists regarding PCa.

The FG sessions, each including 6–8 GPs and/or urologists, were conducted via Zoom, based on the participants’ availability, from February to April 2023, and lasted 45–60 min. The sessions were conducted until information saturation was achieved for each research question. Before each session, the moderator (C.L.) reminded the participants of the study goals and recorded the sessions to allow for posterior analysis. The moderator also ensured the confidentiality of the FG content and that data would be processed without identifying participants. Participation was voluntary and without incentives, and all participants gave their explicit written informed consent to participate in this study and to have the discussion recorded. All participants received a certificate of participation. Prior to the study, participants had only initial contact with the researchers regarding the study’s conduct and overall project goals.

The FG sessions followed the methodology alignment of previous studies [21,22,23], the Consolidated Criteria for Reporting Qualitative Research (COREQ) (see Supplementary File, Table S1) [24], and a topic guide with several open-ended questions (see Supplementary File, Table S2), based on a previous literature review [5,25]. The moderator intervened to ask questions from the guide when a topic was not directed or if the discussion reached saturation. Field notes were made by a PhD student (M.E.) during the FG sessions to aid in data interpretation.

### 2.4. Analysis

All FG sessions were transcribed verbatim by an independent Ph.D. student (C.L.) to ensure an accurate and detailed recording of the participants’ responses. The transcripts were then coded by another independent Ph.D. student (M.E.) using the acronym FG, followed by an alphanumeric character to label and organize the data. Both C. L. and M. E. independently read and reviewed the transcripts multiple times to ensure the reliability and accuracy of the transcripts and coding process. This process included a comparison of the transcripts and their coding to resolve any discrepancies and confirm their consistency.

LiquidText^TM^ 2.7.50 software was used for data assimilation, as it facilitated the organization, annotations, and in-depth analysis of the transcripts, aiding in the effective management of the data. Subsequently, a theoretical thematic analysis was conducted to identify and interpret themes related to the research questions. This analysis was guided by a framework based on previous studies [21,26,27]. During the analysis, codes were generated based on key elements and recurring patterns found in the data. After generating these initial codes, they were organized and grouped into broader themes, which were defined according to their relevance to the research questions and theoretical framework. Subsequently, subthemes emerged from the main themes, offering a more detailed and structured presentation of data. In cases of disagreement, a third researcher (F.R.) served as a referee to reach an agreement.

## 3. Results

### 3.1. Participants

Three FG sessions, involving 21 participants, were conducted until information saturation was reached. Of the 21 participants, 11 (52.4%) were women (Table 1). The median age of all participants was 30.7, with the youngest participant being 25 years old and the oldest being 48. Regarding medical specialties, only one participant was a urologist and the remaining participants were GPs. Participants had an overall 4.8 ± 4.26 years of experience (n = 19) and an average of 375.7 ± 122.34 appointments per month (n = 11). Of the 24 individuals invited to participate, only three declined due to unavailability.

### 3.2. Themes and Subthemes

As mentioned in the Materials and Methods section, a thematic analysis was undertaken, resulting in seven themes: (i) perceptions of PCa, (ii) perceptions of PCa risk factors, (iii) the nutritional impact on PCa, (iv) the role physical activity in PCa, (v) alcohol consumption and smoking, (vi) sexual activity and STDs, and (vii) screening, diagnosis and treatment methods. Each theme has at least two subthemes, which can be observed in Table 2 (see quote examples for each subtheme in the Supplementary File, Table S3). The main highlights of the FG sessions can be found at the end of the Results section (Table 3).

### 3.3. Perceptions about Prostate Cancer

**Figure 1:**
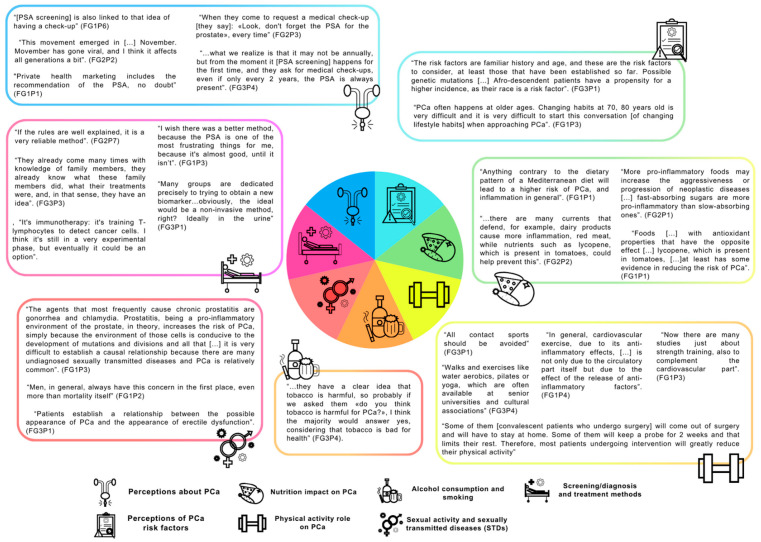
Schematic representation of themes and quotes.

In all FGs, there was a consensus that there is an increasing concern among men regarding PCa, with many seeking PSA screening as a preventive measure (Figure 1).

*“[PSA screening] is also linked to that idea of having a check-up”.*

(FG1P6)

*“When they come to request a medical check-up [they say]: «Look, don’t forget the PSA for the prostate», every time”.*

(FG2P3)

They believe that this might be explained by private health marketing and awareness campaigns, such as Movember (an awareness campaign carried out in November aiming to raise awareness about male diseases, with an emphasis on the prevention and early diagnosis of PCa):

*“Private health marketing includes the recommendation of the PSA, no doubt”.*

(FG1P1)

*“This movement emerged in […] November. Movember has gone viral, and I think it affects all generations a bit”.*

(FG2P2)

The age of first screening starts around 40–50 years, mostly because they (1) consider it is around that age that they should receive the first screening, (2) have a family history, or (3) have knowledge of someone who has or has had PCa. After the first screening, patients usually undergo annual screening or screening every two years.

*“…what we realize is that it may not be annually, but from the moment it [PSA screening] happens for the first time, and they ask for medical check-ups, even if only every 2 years, the PSA is always present”.*

(FG3P4)

They also felt that socioeconomic factors or age could influence PCa-associated health literacy, because younger patients would also look for information on the Internet. The symptoms of PCa were discussed, highlighting that although the initial stages of PCa do not present any symptoms, patients are aware of them, despite their being common in benign prostatic hyperplasia (BPH).

### 3.4. Perceptions of Prostate Cancer Risk Factors

The primary PCa risk factors identified by all FG participants were age and family history, genetic mutations and black race also being mentioned (Figure 1):

*“The risk factors are familiar history and age, and these are the risk factors to consider, at least those that have been established so far. Possible genetic mutations […] Afro-descendant patients have a propensity for a higher incidence, as their race is a risk factor”.*

(FG3P1)

Although lifestyle factors such as diet, exercise, alcohol consumption, smoking, and stress may contribute to PCa development, participants also defended the idea that further studies are necessary to establish a strong correlation, as this has not been fully explored. They also stated that their patients only perceived age and family history as the main risk factors; for this reason, it may be difficult for them to make lifestyle changes to reduce PCa risk, especially at older ages:

*“PCa often happens at older ages. Changing habits at 70, 80 years old is very difficult and it is very difficult to start this conversation [of changing lifestyle habits] when approaching PCa”.*

(FG1P3)

### 3.5. Nutritional Impact on Prostate Cancer

The nutritional impact on PCa was also discussed (Figure 1), in which two subthemes arose: types of diets to aggravate PCa and types of diets to prevent PCa. One participant stated the following:

*“Anything contrary to the dietary pattern of a Mediterranean diet will lead to a higher risk of PCa and inflammation in general”.*

(FG1P1)

*“More pro-inflammatory foods may increase the aggressiveness or progression of neoplastic diseases […] fast-absorbing sugars are more pro-inflammatory than slow-absorbing ones”.*

(FG2P1)

There was also some debate about the impact of specific foods on this type of cancer, and some participants suggested that dairy products and red meat may be pro-inflammatory, which could consequently promote PCa progression:

*“…there are many currents that argue, for example, dairy products cause more inflammation, red meat as well, while nutrients such as lycopene, which is present in tomatoes, could help prevent this”.*

(FG2P2)

*“Foods […] with antioxidant properties that have the opposite effect [...] lycopene, which is present in tomatoes, […] at least has some evidence in reducing the risk of PCa”.*

(FG1P1)

All focus group participants seemed to agree that a healthy diet could be beneficial in reducing the risk of developing PCa or at least help patients endure their treatments and achieve better outcomes. However, they also acknowledged that changing dietary habits could be challenging, especially for older adults who have already established dietary patterns.

### 3.6. Physical Activity Role in Prostate Cancer

In this theme, three subthemes arose: Physical Activity (PA) recommendation and prescription, types of PA to prevent PCa, and willingess to become more active. All participants indicated that they always recommended and/or prescribed exercise to their patients during appointments, depending on their available time (Figure 1). Regarding the types of PA that could be helpful:

*“In general, cardiovascular exercise, due to its anti-inflammatory effects, […] is not only due to the circulatory part itself but due to the effect of the release of anti-inflammatory factors”.*

(FG1P4)

*“Now there are many studies just about strength training, also to complement the cardiovascular part”.*

(FG1P3)

*“Walks and exercises like water aerobics, pilates or yoga, which are often available at senior universities and cultural associations”.*

(FG3P4)

*“All contact sports should be avoided”.*

(FG3P1)

However, it was also mentioned that *“Some of them [convalescent patients who undergo surgery] will come out of surgery and will have to stay at home. Some of them will keep a probe for 2 weeks, which limits their rest. Therefore, most patients undergoing intervention will greatly reduce their physical activity.”* Participants also revealed that their patients were unlikely to become more active, this being associated with their depressive state after diagnosis.

### 3.7. Alcohol Consumption and Smoking

The impact of alcohol exposure on PCa, the impact of smoking exposure on PCa, and patients’ perceptions were the three subthemes identified in this section. Although all participants acknowledged that alcohol consumption, smoking, and exposure to environmental pollutants have a detrimental impact on overall health, potentially contributing to oxidative stress, which could negatively affect PCa, they also highlighted that no strong causal relationship was identified (Figure 1). Participants noted that there was no clear association between tobacco and alcohol consumption, and most mentioned that their patients would not make any correlation:

*“…they have a clear idea that tobacco is harmful, so probably if we asked them «do you think tobacco is harmful to PCa?», I think the majority would answer yes, considering that tobacco is bad for health”.*

(FG3P4)

### 3.8. Sexual Activity and Sexually Transmitted Diseases

The impact of sexual activity on PCa and vice versa were the two subthemes identified. Some participants considered that STDs could be a risk factor for PCa, despite the lack of a strong established relationship between STDs and PCa (Figure 1):

*“The agents that most frequently cause chronic prostatitis are gonorrhea and chlamydia. Prostatitis, being a pro-inflammatory environment of the prostate, in theory, increases the risk of PCa, simply because the environment of those cells is conducive to the development of mutations and divisions and all that […] it is very difficult to establish a causal relationship because there are many undiagnosed sexually transmitted diseases and PCa is relatively common.”.*

(FG1P3)

Participants also emphasized that sexual performance is a major concern for men with PCa. Consequently, some men often delay their medical appointments until they experience symptoms of sexual impotence. Thus, they pursue alternative treatments because of concerns about the side effects of these treatments.

*“Men, in general, always have this concern in the first place, even more than mortality itself”.*

(FG1P2)

*“Patients establish a relationship between the possible appearance of PCa and the appearance of erectile dysfunction”.*

(FG3P1)

### 3.9. Screening/Diagnosis and Treatment Methods

Regarding screening and diagnosis methods, some participants mentioned that PSA is the best available screening method if patients comply with the recommendations. Others have stated that, while PSA is the best current method, it is not as effective as desired (Figure 1):

*“If the rules are well explained, it is a very reliable method”.*

(FG2P7)

*“I wish there was a better method, because the PSA is one of the most frustrating things for me, because it’s almost good until it isn’t”.*

(FG1P3)

They also stated that most patients only know PSA as a screening method, although some are aware of ultrasound scans and digital rectal examination (DRE). They also mentioned that many patients have pre-existing knowledge about PCa and its treatment from family members:

*“They already come many times with knowledge of family members, they already know what these family members did, what their treatments were, and, in that sense, they have an idea”.*

(FG3P3)

Furthermore, they highlighted the potential side effects of PCa treatment, such as urinary incontinence, urinary urgency, diastasis of the rectum, sexual dysfunction, tiredness, and weakness. Despite these side effects, none of the participants reported that any patient abandoned their treatment when prostatectomy was not the selected choice.

Most participants emphasized that it would be important to find alternative non-invasive screening/diagnosis methods, as they underlined that a new biochemical biomarker, especially present in urine, could be the solution:

*“Many groups are dedicated precisely to trying to obtain a new biomarker…obviously, the ideal would be a non-invasive method, right? Ideally in the urine”.*

(FG3P1)

Finally, regarding alternative treatments, one participant stated, *“It’s immunotherapy: it’s training T-lymphocytes to detect cancer cells. I think it’s still in a very experimental phase, but eventually it could be an option.”*

**Table 3:** Main highlights from FG sessions.

Perceptions about PCa	Patients have a **basic understanding** of PCa, its primary screening method, and at what age they should undergo screening
	Men are **more likely** to request screening at **earlier stages** if they have **family or friends with PCa**
	Heightened awareness can be due to private health marketing and awareness campaigns (Movember)
	Men > 75 years should not undergo PSA screening unless they exhibit **symptoms** or have important **comorbidities**, and a life expectancy above 10–15 years
	Patients are aware of benign prostatic hyperplasia (BPH) symptoms but **earlier stages of PCa do not present symptoms**
	Patients undervalue PCa symptoms as they usually link them to other health conditions
	Socioeconomic factors affect PCa health literacy
Perceptions about PCa risk factors	Age, family history, genetic alterations, and black race are the **most recognized risk factors**
	Lifestyle factors are considered **possible contributors** to PCa development
	Health professionals do not expect their patients to adopt lifestyle changes, especially at **older ages**
	Several qualitative studies demonstrate that when facing a PCa-positive diagnosis, **men tend to adopt healthier lifestyles**, especially if advised by their GP
	If patients were advised to alter their habits, they could prevent PCa or, at least, achieve better treatment outcomes.
Perceptions on the association between lifestyle and PCa	Diets rich in **vegetables (tomatoes) and fruits,** and low in **saturated fats and fast-absorbing carbohydrates,** are judged to reduce PCa risk and are recommended by the participants
	After facing a positive PCa diagnosis, men tend to avoid **processed food, red meat, and salt intake** and improve the **consumption of green tea, olive oil, fruits, and vegetables, mainly tomatoes and broccoli** (Mediterranean patterns)
	**Cardiovascular exercise,** like brisk walking, water aerobics, pilates, and yoga are recommended for older patients, while **contact sports** should be avoided
	Older men would be more motivated to engage in **physical activities** if they received **exercise support**
	Exposure to **alcohol** and **tobacco** at younger ages could promote oxidative stress and negatively affect PCa progression
	PCa’s effect on **sexual performance** has become a major concern for men, resulting in screening avoidance until symptoms of **sexual impotence emerge**
Screening/diagnosis and treatment methods	PSA is the best available screening method when patients comply with the recommended precautions, but should be performed along with DRE
	PSA has remained a **controversial** screening method due to its **limitations, overdiagnosis, and overtreatment**
	Need for new **biochemical biomarkers**, highlighting the necessity of **urine biomarkers** as potential non-invasive screening/diagnostic tools for PCa
	PCa treatments often lead to urinary incontinence, urinary urgency, rectal diastasis, sexual dysfunction, fatigue, and weakness
	Alternative treatment methods include an accurate imaging method or the promising field of **immunotherapy** and **brachytherapy** (the application remains experimental)

## 4. Discussion

### 4.1. Perceptions about Prostate Cancer

Our results imply that all FG participants agreed that their male patients have a basic understanding of PCa, its primary screening method, and the age at which they should undergo screening (Table 3). Men who have a friend, neighbor, or family member with PCa are more likely to request screening at an earlier stage, which is confirmed by studies that suggest that social relationships influence men’s decisions to be tested for PCa [28,29]. Additionally, younger men might exhibit the same behavior if they research the disease online, as screening is easily available as a blood test. However, heightened awareness of PCa screening could also be attributed to private health marketing and awareness campaigns such as Movember [30]. Subsequent screenings are typically performed annually or every two years, mostly because patients believe that it should be performed as a checkup. Men over 75 years old should not undergo PSA screening unless they show symptoms [31,32]. This is because only approximately half of the men in this age group are expected to live for another 10 years, and screening is unlikely to be beneficial for older men with other significant health issues. Nevertheless, a recent study suggested that healthy men over 70 years old could continue PSA screening if they do not have any important comorbidities and have a life expectancy of 10–15 years [33]. It is therefore important to reinforce these recommendations. Regarding how PCa symptoms are perceived by patients, while most participants emphasized that patients are undoubtedly aware of BPH symptoms, which are similar to PCa, one participant noted that these symptoms are not present in the early stages of the disease, but only in extremely advanced stages of PCa. Furthermore, BPH symptoms may increase the detection rate of asymptomatic PCa by promoting additional medical appointments and urological examinations. Additionally, more than 20% of men with PCa were found to also have BPH [34]. However, according to other studies, patients may not always associate the symptoms with the disease, as they often devalue them, attributing them to other health conditions such as hypertension, or not considering them a medical issue. It has also been reported that men usually become more aware of symptoms if there is someone in the family with PCa [35,36]. The influence of socioeconomic factors on PCa knowledge was also discussed, as many participants agreed that lower socioeconomic status affects health literacy [37], while others stressed that a younger age or family history are the reasons people could be more aware of the impact of PCa.

### 4.2. Perceptions of Prostate Cancer Risk Factors

Age, family history, genetic alterations, and black race were identified as the primary risk factors for PCa, with the first two being the only ones recognizable by patients. These factors are well established [38,39,40], whereas certain lifestyle factors, such as diet [41,42], lack of exercise [43], alcohol consumption [44,45], and tobacco use [46] were also considered potential contributors to PCa development (Table 3). Even in other studies, some men do not perceive lifestyle as a significant risk factor, partly because their doctors do not advise them to change their habits, and there is no strong evidence that demonstrates that a specific diet or physical activity could prevent PCa [12,17,47].

However, as these are not well established, further studies are necessary to identify PCa-specific risk factors associated with lifestyle. Nevertheless, healthcare professionals do not expect their patients to adopt lifestyle changes, particularly at older ages. An Italian cross-sectional study found that while many men who were diagnosed with PCa were not sufficiently active, they were also unwilling to alter their lifestyle choices, which may indicate a cultural problem [48]. Moreover, a qualitative study showed that some men do not regularly read scientific papers and would only change their lifestyle if advised by their GP [17]. Health professionals also exhibit contradictory behaviors; some recommend dietary and physical activity alterations, while others believe that lifestyle alterations will not benefit frail patients, as there are no guidelines specifically to prevent PCa [49]. Nevertheless, several other qualitative studies support that men change their lifestyle when facing a positive PCa diagnosis [12,50,51,52], often motivated by family and friends [12]. This suggests that if patients are advised to alter some of their habits beforehand, it could help in preventing the disease or, at the very least, achieve better outcomes during treatment.

### 4.3. Perceptions of the Association between Lifestyle Factors and Prostate Cancer

Concerning the impact of nutrition on PCa, a pro-inflammatory diet or a non-Mediterranean diet, dairy products, red meat, and rapidly absorbed sugars could lead to an increase in inflammation and, consequently, to neoplastic disease progression, although the literature remains controversial [53,54,55]. A recent study showed that a higher intake of foods representative of the Western diet, such as high-fat dairy products, red and processed meats, refined grains, sweets, caloric drinks, convenience foods and sauces, should be reduced to prevent this disease [56]. Foods with antioxidant properties, such as lycopene, found in tomatoes, were also found to decrease PSA concentrations and DNA damage, thereby reducing PCa risk [57,58,59]. Additionally, diets rich in vegetables and fruits, and low in saturated fats and fast-absorbing carbohydrates, were recommended by the participants. Studies have reported that after being diagnosed, men tend to avoid processed food, fast food, red meat, and salt intake, while increasing the consumption of green tea, olive oil, fruits, and vegetables, mainly tomatoes and broccoli, aligning with a Mediterranean diet [12,17,50,51,60] (Table 3). However, it has also been argued that since there is no proven link between nutrition and PCa development, there are no reliable studies that demonstrate how any food could promote or prevent PCa progression. Moreover, all participants agreed that they had never seen a study that could support a link between coffee consumption and PCa, despite a systematic review and meta-analysis conducted in 2021 suggesting that increased coffee consumption could be associated with a reduced risk of PCa [61].

Regarding the impact of PA on PCa, although all FGs believed that it could improve the quality of life of patients with PCa, its impact on PCa progression remains controversial. Participants referred to several studies with different results, which could make it extremely difficult to define a specific PA type directed to PCa. Indeed, a review from 2017 also reported inconsistent results, since approximately half of the studies showed that PA was associated with a significant PCa risk reduction of up to 30%, 31 studies reported no association, and only seven of them described an increased PCa incidence [62]. However, cardiovascular exercise was highlighted for its anti-inflammatory effects [63], and strength training was also considered beneficial in a general way. Exercises such as walking [64], water aerobics, pilates, and yoga [65] were recommended for older patients, while contact sports were to be avoided, as most participants emphasized that the best workout should be the most accessible and customized for each patient. However, participants recognized that individuals undergoing surgery or other interventions might have reduced physical activity and muscle loss during recovery, despite enhanced therapeutic results, improved quality of life [66], and increased self-esteem [67]. A qualitative study that addressed the attitudes and preferences of men on active surveillance for PCa regarding exercise and exercise-based support [52] reported that especially older men would be interested and more motivated to engage in physical activities if they received exercise support, as they could feel more in control of PCa outcomes. Therefore, this could be an alternative for men who are more reluctant or have more difficulty in becoming more active.

Participants agreed that drinking alcohol, smoking, and exposure to pollutants negatively affected their general health. However, regarding their impact on PCa, participants noted that establishing a strong association is challenging compared to the one seen in bladder or lung cancer for smoking habits [68,69]. Exposure to alcohol or smoking at younger ages could be a significant factor for oxidative stress and negatively affect PCa progression, as observed in other studies [70,71]. Therefore, they believed that their patients would only acknowledge the negative aspects of these behaviors without directly connecting them to PCa. Participants stated that red wine is more likely to have a protective effect against cardiovascular diseases or in the context of social and mental health than specifically against PCa. Given the anti-inflammatory and antioxidant properties of its flavonoid content, red wine has been reported to have anticarcinogenic properties [72]. However, additional long-term clinical studies are required to establish a definitive correlation between these risk factors and PCa [73].

Regarding sexual activity and STDs, while some participants believed that certain STDs could create a pro-inflammatory environment that increases the risk of PCa, they recognized the challenge in establishing a strong relationship due to the prevalence of undiagnosed STDs. According to a meta-analysis, men with gonorrhea had a 20% increased risk of developing PCa, and syphilis was significantly associated with PCa risk [74]. Moreover, gonorrhea and human papillomavirus (HPV) were strongly associated with PCa risk in another study [75]. Therefore, gonorrhea may be a potential risk factor; however, further studies are necessary to prove a potential association.

In contrast, the effect of PCa on sexual performance has become a major concern for men, often leading to screening avoidance until symptoms of sexual impotence emerge [76,77]. Participants also believed that this concern could be cultural, as patients above 80 years old might still be worried about sexual dysfunction. Thus, all participants agreed that patients typically link impaired sexual performance with PCa, either as a symptom or as a treatment side effect, as confirmed by Zaider [78].

### 4.4. Perceptions of the Screening/Diagnosis and Treatment Methods

In terms of screening and diagnostic methods, participants had varying opinions on the effectiveness of PSA screening. While some considered it the best available screening method when patients comply with the recommended precautions, others expressed reservations and emphasized the need for alternative non-invasive screening/diagnostic approaches (Table 3).

A qualitative study with FGs conducted in Norway revealed that GPs were frequently uncertain whether to recommend PSA screening for patients without a family history of PCa. Additionally, they were unsure of how to interpret the results and when to refer patients for further examinations [79]. PSA has remained a controversial screening method due to its limitations, overdiagnosis, and overtreatment [80,81].

Regarding DRE, most GPs highlighted that many of their colleagues do not usually perform this exam; however, those who do note that if abnormalities in the prostate are detected, urologists often value the results of DRE more than those from PSA [81]. However, they emphasized that both should be performed rather than one. Nonetheless, in a national survey of the Portuguese population, 13.8% of the participants reported having been screened only through DRE, 12.2% only through PSA testing, and 18.2% with both tests, indicating that these PCa screening methods are similarly used [82].

The potential side effects of PCa treatments, such as urinary incontinence, urgency, rectal diastasis, sexual dysfunction, fatigue, and weakness, were acknowledged, which was also confirmed in other studies [83,84].

Finally, when discussing alternative screening or diagnostic methods, the participants expressed the need for new biochemical biomarkers, highlighting the potential of urine biomarkers as potential non-invasive screening/diagnostic tools for PCa [85,86]. An accurate imaging method would also be a good alternative, with the most important screening or diagnosis method being one that is cost-effective and has high specificity and sensitivity. Additionally, one participant mentioned the promising field of immunotherapy and brachytherapy, although its application in PCa remains experimental [87,88,89].

### 4.5. Limitations and Strengths

Our study has some limitations. The small sample size of 21 participants across the three FGs may limit the generalizability of the findings. Additionally, the limited number of urologists among the participants may restrict the diversity of perspectives within this specialized field. Their inclusion could provide valuable insights and complement the viewpoints offered by GPs, thereby enhancing the overall depth of the study. Despite these constraints, in qualitative research, the number of participants is not relevant, as FGs aim to explore diversity and determine the range of views rather than establish representativeness [90] Moreover, the qualitative nature of our research allowed us to achieve data saturation, providing a comprehensive understanding of key themes related to health professionals’ views on PCa in this regional context. This depth of insight underscores the value of our findings even with a smaller sample size. Furthermore, a systematic review from 2022 found FG studies with widely varying numbers of participants and focus groups, ranging from one to 40 FGs, with one study not reporting these data at all [91]. Additionally, this review emphasizes that achieving data saturation, rather than the number of participants, is the key determinant of qualitative research quality. This review found that saturation is typically reached with four to eight focus groups, particularly in studies with relatively homogeneous populations and well-defined objectives. This finding aligns with our study design and supports the adequacy of our sample size.

We employed a snowball sampling strategy to recruit health professionals for the FG sessions. While this approach can introduce selection bias by potentially leading to a sample with similar backgrounds or perspectives, we attempted to mitigate this bias by employing additional recruitment methods, such as contacting health centers and conducting multiple waves of recruitment. Despite these efforts, some degree of selection bias may still exist.

Our study was conducted online, as previously mentioned. While online FG sessions present some challenges, such as the lack of personal connection, which might make some participants less engaged or forthcoming, and potential technical issues, they also offer several advantages [92]. An online FG reduces geographical limitations (the region covered by our study, the central region of Portugal, covers more than 23,000 km^2^), increases scheduling flexibility, and lowers costs associated with travel, venue hire, and logistics. Additionally, digital recordings and transcripts facilitate data collection, management, and analysis. Moreover, participants may feel more comfortable and relaxed in their own environment, which can lead to more open and honest responses [93,94].

Despite these limitations, our study provides an in-depth exploration of healthcare professionals’ perceptions and experiences regarding risk factors, screening practices, and the impact of lifestyle factors. Although this study did not include patients, it sheds light on their major concerns, particularly regarding their sexual performance and treatment side effects. The outcomes of this qualitative study will be useful in the design of a questionnaire to be applied to the male population to identify differences in lifestyles, and potentially establish a correlation between lifestyle factors and PCa. Given the increasing incidence of PCa, we believe our study may contribute to future research and aid in designing interventions and strategies aimed at improving urological health outcomes and provider education.

## 5. Conclusions

The findings of this study provide valuable insights into the perceptions and experiences of PCa among healthcare professionals. To the best of our knowledge, this is the first qualitative study to explore the awareness and experience of these professionals, comprising several aspects of the disease, from the general perception of PCa to risk factors, screening, diagnosis, and treatment. This study highlighted that healthcare professionals recognize advanced age and family history as significant PCa risk factors and emphasize the promotion of healthier lifestyles for their patients, despite some conflicting views on lifestyle factors. While further research is needed to establish a correlation between lifestyle factors and PCa development, our study underlines that healthcare professionals can promote healthier lifestyles to their patients. Increasing evidence suggests that men are willing to improve their habits, such as increasing vegetable consumption (e.g., tomatoes) and engaging in physical activity, which can be easily integrated into patients’ lives. Moreover, further research into non-invasive screening methods, such as biomarkers and alternative treatment options, is required, to prevent false positives and treatment side effects. Finally, given the important role that healthcare professionals play, educational and intervention strategies could be valuable for both professionals and patients in raising awareness about this cancer.

## Figures and Tables

**Table 1:** Participants’ characteristics.

Characteristics	n	Percentage (%)
Total Participants	21	100%
**Gender**		
Men	10	47.6%
Women	11	52.4%
**Age**		
18–29	11	52.4%
30–39	7	33.3%
40–49	2	9.5%
**Medical Specialty**		
GPs	20	95.2%
Urologists	1	4.8%
**Professional experience**	**n**	**Mean ± SE**
Years of experience	19	4.8 ± 4.26
Appointments per month	11	375.7 ± 122.34

**Table 2:** Themes and subthemes from FG sessions.

Theme	Subtheme
Perceptions about prostate cancer (PCa)	Age at first PCa screening and frequency
	Symptoms
	Impact of socioeconomic factors
	Reasons for PCa screening
Perceptions of PCa risk factors	Risk factors acknowledged by participants
	Patients’ knowledge about risk factors
	Willingness to change lifestyle habits
Nutritional impact on PCa	Diets that aggravate PCa
	Diets that prevent PCa
	Supplementation prescription
	Willingness to change diets
Physical activity (PA) role in PCa	PA recommendation and prescription
	PA to prevent PCa
	Willingness to become more active
Alcohol consumption and smoking	Young-age exposure to alcohol
	Young-age exposure to smoking
	Patients’ perceptions
Sexual activity and sexually transmitted diseases (STDs)	Impact of sexual activity on PCa
	Impact of PCa on sexual performance
Screening, diagnosis, and treatment methods	Screening/diagnosis methods perceptions
	Alternative diagnosis methods
	Treatment methods perceptions
	Alternative treatment methods

## Data Availability

The original contributions presented in the study are included in the article and Supplementary Materials; further inquiries can be directed to the corresponding author/s.

## Supplementary Materials

The following supporting information can be downloaded at: https://www.mdpi.com/article/10.3390/cancers16173005/s1, Table S1: Consolidated criteria for reporting qualitative studies (COREQ): 32-item checklist; Table S2: Guide for focus groups sessions; Table S3: Thematic analysis results with quote examples.
