# Supplementary material for: Health Professionals’ Perceptions about Prostate Cancer—A Focus Group Study
Source: Cancers (Basel). 2024 Aug 29;16(17):3005. doi: 10.3390/cancers16173005 (PMC11394291; doi:10.3390/cancers16173005)
Supplement: Supplementary file 1 [file cancers-16-03005-s001.zip › cancers-3113306-supplementary.pdf]

Supplementary Materials

Table S1. Consolidated criteria for reporting qualitative studies (COREQ): 32-item checklist.

No	*Item*	*Guide questions/description*	*Check*
Domain 1: Research team and reflexivity			
Personal Characteristics			
1	Interviewer/facilitator	Which author/s conducted the interview or focus group?	Pg. 3, Section 2.3
2	Credentials	What were the researcher's credentials? E.g. PhD, MD	Pg. 3, Section 2.4
3	Occupation	What was their occupation at the time of the study?	Pg. 3, Section 2.4
4	Gender	Was the researcher male or female?	Female
5	Experience and training	What experience or training did the researcher have?	Pg. 3, Section 2.3
Relationship with participants			
6	Relationship established	Was a relationship established prior to study commencement?	Pg. 3, Section 2.3
7	Participant knowledge of the interviewer	What did the participants know about the researcher? e.g. personal goals, reasons for doing the research	Pg. 3, Section 2.3
8	Interviewer characteristics	What characteristics were reported about the interviewer/facilitator? e.g. Bias, assumptions, reasons and interests in the research topic	Pg. 3, Section 2.3
Domain 2: study design			
Theoretical framework			
9	Methodological orientation and Theory	What methodological orientation was stated to underpin the study? e.g. grounded theory, discourse analysis, ethnography, phenomenology, content analysis	Pg. 3, Section 2.3
Participant selection			
10	Sampling	How were participants selected? e.g. purposive, convenience, consecutive, snowball	Pg. 3, Section 2.2
11	Method of approach	How were participants approached? e.g. face-to-face, telephone, mail, email	Pg. 3, Section 2.2
12	Sample size	How many participants were in the study?	Pg. 3, Section 2.3
13	Non-participation	How many people refused to participate or dropped out? Reasons?	Pg. 3, Section 2.3
Setting			
14	Setting of data collection	Where was the data collected? e.g. home, clinic, workplace	Workplace, via Zoom conference
15	Presence of non-participants	Was anyone else present besides the participants and researchers?	No
16	Description of sample	What are the important characteristics of the sample? e.g. demographic data, date	Pg. 4, Table 1
Data collection			
17	Interview guide	Were questions, prompts, guides provided by the authors? Was it pilot tested?	No
18	Repeat interviews	Were repeat interviews carried out? If yes, how many?	No
19	Audio/visual recording	Did the research use audio or visual recording to collect the data?	Pg. 3, Section 2.3
20	Field notes	Were field notes made during and/or after the interview or focus group?	Pg. 3, Section 2.3
21	Duration	What was the duration of the interviews or focus group?	Pg. 3, Section 2.3
22	Data saturation	Was data saturation discussed?	Pg. 4, Section 3.1
23	Transcripts returned	Were transcripts returned to participants for comment and/or correction?	No
Domain 3: analysis and findings			
Data analysis			
24	Number of data coders	How many data coders coded the data?	Pg. 3-4, Section 2.4
25	Description of the coding tree	Did authors provide a description of the coding tree?	Pg. 4-5, Table 2
26	Derivation of themes	Were themes identified in advance or derived from the data?	Pg. 4-5, Section 2.4
27	Software	What software, if applicable, was used to manage the data?	Pg. 4, Section 2.4
28	Participant checking	Did participants provide feedback on the findings?	No
Reporting			
29	Quotations presented	Were participant quotations presented to illustrate the themes/ findings? Was each quotation identified? e.g. participant number	Pg. 5- 8, Section 3.3 – 3.9
30	Data and findings consistent	Was there consistency between the data presented and the findings?	Yes

31	Clarity of major themes	Were major themes clearly presented in the findings?	Pg. 4-8, Table 2, Section 3.3 – 3.9
32	Clarity of minor themes	Is there a description of diverse cases or discussion of minor themes?	Pg. 4, Table 2

Table S2. Guide for focus groups sessions.

Kreugers' categories	*Questions*
Opening question	Please indicate your age, specialty, how many years you have been working, and how many patients you have and have an appointment with you per month.
Introductory questions	Do you think your patients know about prostate cancer and is it a disease that worries them? At what age does that usually happen?
	Do you think your patients know PCa symptoms?
	Do you believe that socioeconomic factors influence PCa-related health literacy?
	Do your patients usually get tested as a check-up or just when they present any symptoms?
Transition questions	Which are the PCa risk factors that are more involved in the progression of the disease? Do you think your patients have the same perception?
	Do you feel that your diagnosed patients are more available to change their lifestyles?
Key questions	From your experience and opinion, is there any food or diet that may potentiate the development of PCa?
	From your experience and opinion, is there any food or diet that could avoid the development of PCa?
	Is it usual for you prescribe any supplements for your PCa patients?
	Do you think that coffee may have a positive role in the development of PCa?
	What is your perception regarding your patients' physical capacity? Is it usual to recommend physical activity to them?
	In your opinion, is there any physical activity (PA) or exercise that you think might influence PCa progression?
	Do your patients become more active when they have a PCa diagnosis?
	In your opinion, does alcohol and tobacco exposition at younger ages potentiate PCa progression?
	Could red wine have a protective role in this disease?
	Which are the tobacco consumption factors that you believe could influence PCa development?
	In what way, do you think that sex life or sexually transmitted diseases affect the development of PCa?
	From your professional experience, do your patients have any perception or concern that PCa may affect their sexual performance?
	Are your patients informed about PCa screening/diagnosis methods?
	Are there any screening/diagnosis methods that you think could be a good alternative or that could be used as a complement to the existing methods?
	Are your patients informed about PCa treatment methods?
	Do you have any knowledge about any side effects that your patients felt during PCa treatment? Is there any possibility for them to abandon the selected treatment?
Ending questions	Based on your knowledge, do you think it is possible to develop better screening/diagnosis methods? In what way could this be important to clinical practice?
	Do you feel like what we talked about, is there anything else that should have been addressed?

Table S3. Thematic analysis' results with quote exemples.

Theme/Subtheme	*Participant*	*Quote*
Perceptions about PCa		
Age of first PCa screening and frequency	FG1P4	*"In their forties, they begin to look for it [PSA screening], and we see men in their 70s-80s still asking to go on surveillance, so it's a very present concern for them."*
	FG3P3	*"When they reach the age of 50, almost all of them come with the idea of "I'm in my 50s, I must see this issue here with the prostate", so I think that if I had to evaluate an age in which there is a greater concern, is effectively from 50 onwards".*
Symptoms of the disease	FG2P1	*"They already know that there are essentially symptoms of the urinary tract, and they are already beginning to have this notion, of waking up many times at night or urinary stream weakness."*
	FG3P1	*"Early-stage prostate cancer has no symptoms, so obviously patients don't know the symptoms, because they are non-existent. And prostate cancers with symptoms are very advanced prostate cancers too."*
Impact of socioeconomic factors	FG1P4	*"I associate it not so much with the socioeconomic part, but more with age groups. It is generally noted that younger people, not that they are better informed, but that they seek more information, perhaps because they have more access to the internet."*
	FG3P6	*"[…] because people who are more literate are more concerned [about their health], they are the ones who go [more frequently] to the doctor."*
Reason for PCa screening	FG2P3	*"The majority who come to us request it [PSA screening] because the neighbor had it and does it recurrently or had prostate cancer […] The rest, I think, they request more when they have complaints."*
	FG3P2	*"Sometimes, when they have a family member, for example, a parent who has had prostate cancer, they anticipate this concern and ask when they should start screening, what should they do, what should they pay attention to."*
Perceptions of PCa risk factors		
Most important risk factors	FG1P3	*"I think prostate cancer was one of the most used cancers as an example for cancers, whose appearance was almost 100% a consequence of age alone, of cell division […] it's tobacco, it's alcohol, it's harmful lifestyle habits, isn't it, stress itself [...] like people who eat more red meat [...] everything that are considered healthy lifestyle habits, are good to avoid cancers in general."*
	FG3P1	*"The risk factors are familiar history and age. And these are the risk factors to consider, at least those that have been established so far. Possible genetic mutations […] We can also consider in our Portuguese population, obviously, that African patients with a propensity for a higher incidence, they have a risk factor increased by the black race, but otherwise, we can't distinguish anything else yet."*
Knowledge of patients about PCa risk factors	FG2P1	*"[…] perhaps the risk factor that they have better perceived will be the genetic factor. Because then yes, there's a history of prostate cancer in the family, it's almost certain that they always come to ask for screening, that's for sure".*
	FG2P5	*"The age, the age they understand"*
Availability of changing lifestyle habits	FG1P3	*"Prostate cancer often happens at older ages, which is the most common age group. Changing habits at 70, 80 years old is very difficult and it is very difficult to start this conversation [of changing lifestyle habits] by approaching prostate cancer".*

	FG3P4	*"Autonomously it is very difficult for them to change. Even for diabetes and hypertension, where there is a clear relationship between lifestyle, diet, physical exercise, and the progression of the disease, it is difficult. I often think that for something that is not so clear to them, it is even more difficult."*
Nutrition impact on PCa		
Types of diets to aggravate PCa	FG1P1	*"Anything that is contrary to the dietary pattern of a Mediterranean diet."*
	FG2P1	*"More pro-inflammatory foods may increase the aggressiveness or progression of neoplastic diseases […] fast-absorbing sugars are more pro-inflammatory than slow-absorbing ones."*
Types of diets to prevent PCa	FG1P1	*"Foods with nutrients, with antioxidant properties that have the opposite effect [...] so the only thing I know, more directed, is lycopene, which is present in tomatoes, […] which at least has some evidence in reducing the risk of prostate cancer."*
	FG2P1	*"Perhaps a diet rich in fruits and vegetables is the best [...] Low in saturated fats and fast-absorbing carbohydrates."*
PA role on PCa		
PA recommendation and prescription	FG1P3	*"This is part of our day-to-day, physical exercise prescription. Therefore, for non-pharmacological measures, we are always recommending the Mediterranean diet and adequate physical activity for the patient."*
	FG3P7	*"We recommend the practice of physical exercise to all patients because of the positive effects it brings at all levels. Of course, it will not be because he has prostate cancer that we are going to increase this incentive because it is already a generalized incentive."*
Types of physical activity to prevent PCa	FG1P4	*"In general, cardiovascular exercise, due to its anti-inflammatory effects, […] is not only due to the circulatory part itself but due to the effect of the release of anti-inflammatory factors."*
	FG3P4	*"The type of exercise that we recommend for this type of patient is more or less the same type of exercise that we recommend for others, namely that which is more accessible and more appropriate, usually for older people [...] walks and exercises like water aerobics, pilates or yoga, which are often even available at senior universities, and cultural associations."*
Availability to become more active	FG1P4	*"The ones I know have either been active before and stayed just as active or honestly I don't see much of a difference."*
	FG3P1	*"Some of them [convalescent patients who undergo surgery] will come out of surgery, will have to stay at home. Some of them will keep a probe for 2 weeks and that limits their rest. Therefore, most patients undergoing intervention will greatly reduce their physical activity."*
Alcohol consumption and smoking		
Impact of exposure to alcohol on PCa	FG1P3	*"Yes. It depends on the type of consumption and the amount and all that, but yes, I'm always going to talk about oxidative stress, but the short answer is yes".*
	FG1P4	*"I would add more, I would say that not only because of the effect of alcohol itself but because of other inadequate lifestyles that are often associated with excessive alcohol consumption."*
Impact exposure to smoking on PCa	FG2P1	*"That's the tobacco load. The greater the smoking load, the greater the risk […] And who says exposure to tobacco, says exposure to environmental pollutants."*
	FG3P1	*"Now, until now, no clear relationship between tobacco consumption and the development of prostate cancer has been identified, nor has it been validated for alcohol consumption, contrary to other carcinomas."*

Patients' perception	FG2P3	*"They do not strongly link these habits to the development of prostate cancer."*
	FG3P4	*"They would probably answer this question if they had to answer yes, because they have a clear idea that tobacco is harmful, so probably if we asked them this question, "do you think tobacco is harmful for prostate cancer?". I think it's the majority, most would answer yes, considering that tobacco is bad for health."*
Sexual activity and STDs		
Impact of sexual activity on PCa	FG1P5	*"Having an STD is a risk factor for getting prostate cancer."*
	FG1P3	*"The agents that most frequently cause chronic prostatitis are gonorrhea and chlamydia. Prostatitis, being a pro-inflammatory environment of the prostate, in theory, increases the risk of prostate cancer, simply because the environment of those cells is conducive to the development of mutations and divisions and all that […] it is very difficult to establish a causal relationship because there are many undiagnosed sexually transmitted diseases and prostate cancer is relatively common."*
Impact of PCa on sexual perception	FG1P2	*"Men, in general, always have this concern in the first place, more than even mortality itself. As a matter of fact, there are many patients who only begin to be monitored [because of this]. I recently had a diabetic [patient] who was never properly monitored until he started to have symptoms of impotence. And like this one, there are many others."*
	FG2P1	*"Maybe not so much with the disease itself and its natural progression […] but they often ask about the side effects of the treatments, and whether or not they can affect the sexual part."*
Screening/Diagnosis and treatment methods		
Screening/ Diagnosis methods perceptions	FG1P3	*"I was saying that in terms of diagnosis, the best diagnosis for prostate cancer is the biopsy and it's hard to come up with anything more specific than that. Sensitivity is worth what it's worth, they don't always hit the most characteristic area of the prostate [...] Now for screening, I wish there was a better method, because the PSA is one of the most frustrating things for me, because it's almost good, until it isn't."*
	FG2P3	*"Yes, in parallel with the PSA, we are often asked for ultrasound scans."*
Alternative screening/ diagnosis methods	FG1P4	*"Maybe finding some biomarker that really allows screening and, eventually, diseases' diagnosis, which nowadays, there are diseases that you can't do the biopsy and diagnose them, like some types of amyloidosis that can be done. A biopsy can be avoided. Now, as the FG1P2 says, what is done on paper, in studies, is one thing, and then moving to real life and being cost-effective goes a long way."*
	FG3P1	*"Many groups are dedicated precisely to trying to obtain a new biomarker. A biochemical marker or possibly an RNA or DNA test will obviously be the way to go. I was talking about PCA3, finding a genetic signature in urine. Obviously, even the ideal would be a non-invasive method, right? Ideally in the urine, many working groups have been devoted to this. And, obviously, this is the Holy Grail for any cancer patient, finding a biomarker with high specificity and high sensitivity."*
Treatment methods perceptions	FG2P1	*"Some young people have already heard about hormone therapy, about seeds, which are rarer cases. Most know that there is surgery, practically everyone knows, and that there is radiotherapy, which has already been explored a lot."*

	FG3P3	*"They already come many times with knowledge from family members, they already know what these family members did, what their treatments were, and, in that sense, they have an idea. They talk a lot about the seeds and that's it, what the family members did, it's more that type of knowledge they have"*
Treatment side effects	FG1P3	*"The prostate is a very sensitive site in terms of muscular and biological terms, so we already know that they have urinary incontinence or urinary urgency"*
	FG3P4	*"Besides sexual dysfunction, right? We already know that tiredness and weakness are negative things."*
Alternative treatment methods	FG1P3	*"It's immunotherapy, it's training T-lymphocytes to detect cancer cells and I think it's still in a very experimental phase, but eventually it would be an option. The most modern thing is brachytherapy, I think, but I'm not really into the therapies for prostate cancer."*
